# Using the Cardiff model to reduce late‐night alcohol‐related presentations in regional Australia

**DOI:** 10.1111/ajr.12983

**Published:** 2023-04-20

**Authors:** Tim Baker, Nicholas Taylor, Kate Kloot, Peter Miller, Diana Egerton‐Warburton, Jonathan Shepherd

**Affiliations:** ^1^ Centre for Rural Emergency Medicine, Faculty of Health Deakin University Warrnambool Victoria Australia; ^2^ South West Healthcare Warrnambool Victoria Australia; ^3^ School of Psychology, Faculty of Health Deakin University Geelong Victoria Australia; ^4^ National Drug Research Institute Curtin University Perth Victoria Australia; ^5^ School of Medicine Deakin University Warrnambool Victoria Australia; ^6^ School of Clinical Sciences at Monash Health Monash University Clayton Victoria Australia; ^7^ Crime and Security Research Institute Cardiff University Wales UK

**Keywords:** alcohol, emergency, public health, rural health, surveys

## Abstract

**Introduction:**

The Cardiff model is a data sharing approach that aims to reduce the volume of intoxicated patients in emergency departments (EDs). This approach has not been tested in a rural setting.

**Objective:**

This study assessed whether this approach would reduce the number of alcohol‐associated presentations during high‐alcohol hours (HAH) in a regional ED.

**Design:**

From July 2017, people over the age of 18 attending the ED were asked by the triage nurse (1) whether they had consumed alcohol in the past 12 h, (2) their typical alcohol consumption level, (3) the location where most alcohol was purchased and (4) the location of the last drink. From April 2018, quarterly letters were sent to the top five venues reported within the ED. Deidentified, aggregated data were shared with local police, licensing authorities and local government, identifying the top five venues reported in the ED and providing a summary of alcohol‐related attendances to the ED. Interrupted time series analyses were used to estimate the influence of the intervention on monthly injury and alcohol‐related ED presentations.

**Findings:**

ITS models found that there was a significant gradual decrease in the monthly rate of injury attendances during HAH (Coefficient = −0.004, *p* = 0.044). No other significant results were found.

**Discussion:**

Our study found that sharing last drinks data collected in the ED with a local violence prevention committee was associated with a small, but significant reduction in the rate of injury presentations compared with all ED presentations.

**Conclusion:**

This intervention continues to have promise for reducing alcohol‐related harm.

1 **What this paper adds**

Our study found that sharing last drinks data collected in the emergency department was associated with a small, but significant reduction in the rate of injury presentations compared with all ED presentations, despite most of the presentations being associated with packaged liquor and drinking at home. The findings of these show that the Cardiff model continues to have promise for reducing alcohol‐related harm, especially if the information networks are formalised and emergency department data can be included in licensing considerations and police proceedings.

2 **What is already known on this topic**

The Cardiff model has been found to be an effective intervention at reducing nightlife alcohol‐related attendances from metropolitan emergency departments. However, until this study, nobody had examined the interventions impact in a regional setting.

## 1 INTRODUCTION

Alcohol‐related harm is one of the most important preventable public health issue impacting providers of emergency care in rural and urban Australia.^1^ A snapshot survey of 106 emergency Australian and New Zealand emergency departments (EDs) found one in seven patients in the ED at that time (a Friday night) was associated with alcohol.^2^ Intoxicated patients delay and disrupt the care of patients around them.^3^ Ninety per cent of emergency clinicians in one survey reported they had experienced physical aggression by intoxicated patients in the previous year.^3^

Intoxicated patients may be particularly disruptive for rural emergency departments. Australians outside metropolitan areas are more likely to drink in excess of recommended guidelines, engage in risky drinking and die from alcohol‐related causes (AIHW 2019). The snapshot survey mentioned above found that the proportion of alcohol‐related attendances is higher in regional emergency departments than suburban departments (and almost as high as major metropolitan departments).^4^ A study from Victoria found that regional emergency departments saw more alcohol‐related injuries than urban emergency departments.^5^ And, in contrast to urban emergency departments, rural emergency care facilities are less likely to have 24‐h onsite security.^6^

The disruption caused by intoxicated patients has motivated some large metropolitan EDs to look for novel solutions. The most studied intervention is the Cardiff model, reported in the UK by Shepherd et al.^7^ This strategy is based on the sharing and use of specific data collected from injured people treated in EDs with public agencies to encourage and guide interventions to reduce alcohol‐related harm. Data collection often concentrated on ED presentations during high‐alcohol hours (HAHs; 8 PM–6 AM Friday and Saturday nights^8^) to be a valid proxy measure of alcohol‐related harm in the community.

This data sharing approach has not been tested in a rural setting.

The aim of this study was to assess whether the driving change approach (based on the public data sharing described above and in our protocol article^9^) would reduce the number of alcohol‐associated presentations during HAH in a regional emergency department.^9^ We hypothesised that this approach would reduce the outcome measures defined as:

- both the absolute number and proportion of presentations during HAHs in which the patient reports alcohol use via the last drinks questions; and
- both the number and proportion of presentations during HAHs assigned an injury diagnosis.

## 2 METHODS

### 2.1 Setting

Warrnambool is a large rural town (Modified Monash Model 3) on the coast of Victoria, Australia. There is an estimated residential population of 35 214.^10^ The Warrnambool entertainment precinct is one of Victoria's 18 designated areas, and this is an area that has experience alcohol‐related violence, within which police have extra powers in order to try and reduce further violence and antisocial behaviour (https://www.vcglr.vic.gov.au/community‐services/government‐initiatives/designated‐areas).

### 2.2 Data

This study used data from a 5‐year study from the National Health and Medical Research Council (NHMRC) *Driving Change* project^9^ (http://lastdrinks.info/).

#### 2.2.1 Presentation data

All people over 18 years old presenting to South West Healthcare Emergency Department (ED) during high‐alcohol hours (HAH) from January 2015 to June 2020 were included in the study. High‐alcohol hours were defined as triage time 8 pm–6 am Friday and Saturday nights. Unfortunately, data from March 2020 and beyond had to be excluded from all analyses due to the unique impact the COVID‐19 pandemic had on ED presentations.

Administrative data from the Victorian Emergency Minimum dataset (https://www.health.vic.gov.au/data‐reporting/victorian‐emergency‐minimum‐dataset‐vemd) was used to determine age, gender, triage time, and primary diagnosis. The patient presentation type was deemed to be an injury if the primary diagnosis was an ICD10 S or T injury code.^11^

### 2.3 Last drinks questions

From July 2017, all people over the age of 18 attending the ED were asked by the triage nurse (1) whether they had consumed alcohol in the past 12 h, (2) their typical alcohol consumption level in a drinking session, (3) the location where most alcohol was purchased and (4) the location of the last drink. Questions were mandatory within the electronic record systems of each ED. Staff were trained on the reasons behind the questions. Clinicians were able to identify when patients were nonresponsive or not willing to ask questions, or when questions were not asked for ethical reasons when the clinician perceived a cultural or interpersonal issue, or other clinical judgement (including the need to treat other patients urgently), that makes the questions inappropriate.^9^ As last drinks data were only available for the final 36 months of the 65‐month project, analyses using this data will be more limited than those examining just injuries.

### 2.4 Intervention

The interventions in the Driving Change project^9^ were based on public health data sharing interventions that were based on the approaches previously trialled and reported in the UK by Shepherd et al.^7^, ^12^, ^13^, ^14^, ^15^ Quarterly letters were sent to the top five venues reported within the ED, outlining the number of attendances related to their business, anonymised details of the cases including diagnoses and anonymised photographs of injuries. Letters were delivered via the Australasian College for Emergency Medicine (ACEM) to registered licensees. Deidentified, aggregated data were also shared with local police, licensing authorities and local government, identifying the top five venues reported in the relevant ED and providing an summary of alcohol‐related attendances to the ED.^9^ Letters were sent to nightlife venues in April 2018, July 2018, November 2018, February 2019 and November 2019.

Also inspired by the previous work conducted in Cardiff,^7^, ^12^, ^13^, ^14^, ^15^ the Warrnambool Violence Prevention Board (WVPB) was set up and aligned with existing community crime prevention initiatives organised by the Department of Justice. The WVPB included hospital staff, police, local health, youth and alcohol treatment services, local council, ambulance and research team representatives. A subgroup of venue operators was set up to align with the local Liquor Accord. The WVPB met quarterly and on an ad hoc basis and regularly raised issues in the media, including a series of 12 articles in print and radio media using project data to highlight local issues and trends.

### 2.5 Analysis

Interrupted time series (ITS) analysis (itsa command) in Stata 15^16^ was used to estimate the influence of the intervention on monthly alcohol‐related ED presentations. ITS allows simultaneous tests of step (immediate) and slope (gradual) changes in trends,^17^ and this allowed us to evaluate the impact of the initial date letters that were sent (step) and the gradual impact from the series of letters (slope). A monthly seasonal variable was included in each model to account for periodic trends. Cross‐correlograms were examined to identify the best‐fitting transfer function for the intervention variable (defined as lag^18^).

Ethics approval was obtained from Deakin University (DUHREC 2017‐178) and the hospital involved in the study (SWHC 05/2017).

## 3 RESULTS

During the HAH in the study period, 6918 patients presented to the emergency department. Table 1 shows the presentation data for the variable used to test the hypotheses set by the protocol article.^9^

**TABLE 1:** Emergency department presentation characteristics, during high alcohol hours.

Analysis	Injuries	Alcohol consumption in past 12 h
Time period	01/01/2015–29/02/2020	01/07/2017–29/02/2020
Yes	1802	549
No	5000	2903
Unknown	116	267
Total	6918	3719

Because the intervention of sending feedback to nightlife venues where injuries were common is only likely to reduce presentations from nightlife venues, we recorded the location of they purchased their last drink. Data from the last drinks survey indicated that 118 of the HAH attendances that had consumed alcohol in the last 12 h came from nightlife venues (see Table 2), this was 22.65% of the 521 cases where location data were available, and 21.49% of all 549 of cases during HAH that had consumed alcohol within the previous 12 h. It was fewer than half the number of presentations where their last drink was at home.

**TABLE 2:** Last drinks data.

Location	Count
*Location of last drink*	
Nightlife	118
Home	262
Other residence	32
Restaurant	11
Other	98
*Location of most drinks purchased*	
Bottle shop	220
Supermarket	181
Nightlife	108
Restaurant	10
Other	27

The majority of cases reported purchasing alcohol from an off‐premise licence (see Table 2). Forty‐eight (40.68%) individuals reported purchasing most drinks from a bottle shop or supermarket before then reported consuming their last drink in a nightlife setting, indicating a substantial proportion of predrinking among ED attendances during HAH.

### 3.1 Time series analysis

#### 3.1.1 Injuries

**FIGURE 1:**
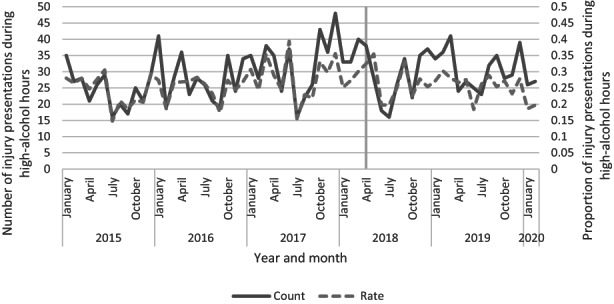
Count and rate of injuries in all presentations during high‐alcohol hours.

Presentations during HAH are a proxy for alcohol‐related harm. We used an ITS model to evaluate the impact of the intervention on the number of injury presentations during HAH. This was examined in two ways: the number of people reporting injuries during HAH and the proportion of injuries during HAH compared with all ED attendances during HAH (Figure 1).

ITS models found that there was a significant slope decrease in the monthly rate of injury attendances during HAH (Table 3).

**TABLE 3:** Injury trends in response to intervention.

Condition		Lag	Coefficient	*p*	CI
Count	**Time (slope)**	1	**0.27**	**0.001**	**0.11, 0.43**
	Intervention (step)		−3.83	0.245	−10.37, 2.71
	Time × Intervention (slope)		−0.30	0.184	−0.74, 0.15
Presentation rate	**Condition**	1	**0.001**	**0.032**	**0.0001, 0.002**
	Intervention (step)		0.00002	0.999	−0.05, 0.05
	**Time × Intervention (slope)**		**−0.004**	**0.044**	**−0.01, −0.0001**

*Note*: Bold text indicates a significant result.

### 3.2 Last drinks questions

**FIGURE 2:**
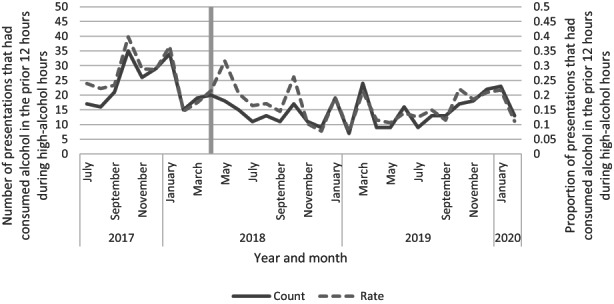
Number of emergency department presentations that reported consuming alcohol in the prior 12 h and the proportion of presentations compared with all presentations during high‐alcohol hours.

We analysed people reporting recent alcohol use as a parallel method of assessing alcohol‐related harm. An ITS model was used to evaluate the impact of the intervention on the number of people reporting alcohol consumption within the last 12 h during HAH. This was examined in two ways: the actual number of people reporting alcohol consumption and the proportion of people reporting alcohol consumption compared to those that reported none (Figure 2).

ITS models found that were no significant changes attributable to the intervention (Table 4).

**TABLE 4:** Last drinks presentation trends in response to intervention.

Condition		Lag	Coefficient	*p*	CI
Count	Time (slope)	4	−0.42	0.617	−2.15, 1.31
	Intervention (step)		−7.04	0.350	−22.49, 8.40
	Time × Intervention (slope)		0.46	0.549	−1.12, 2.03
Proportion of Yes to No	Condition	2	−0.01	0.429	−0.03, 0.01
	Intervention (step)		−0.04	0.662	−0.22, 0.14
	Time × Intervention (slope)		0.005	0.535	−0.01, 0.02

## 4 DISCUSSION

We assessed the impact of the last drinks data sharing intervention by two parallel methods: the number of injuries presenting during HAH and the number of presentations reporting recent alcohol consumption presenting during HAHs. Our study found that the intervention was associated with a small reduction in the rate of injury presentations compared with all ED presentations. It showed no significant impact on the number of people reporting recent alcohol consumption during HAHs, although this has been previously shown as a less reliable measure over time.^8^, ^19^ The discrepancy between the two results may be that the injury protocol had more power to detect significant results, as it was able to examine cases dating back to 2015.

The finding that the intervention was associated with a statistically significant decrease in alcohol‐related presentations is in line with previous research.^7^ Droste et al.^7^ found this intervention to be the most effective in terms of emergency department‐based interventions, and this trial provides some support for the previous findings, although the small effect size is likely due to the small nature of the nightlife setting (pop 25 000). While police were active partners in the WVPG and chaired the Liquor Accord, no specific interventions were introduced into venues which were found to have high violence rates beyond the general awareness raising with venues and media awareness campaign. It is possible that a more structured intervention with clear steps of escalation for violence levels (e.g. mandatory ID scanners for more than 2 assaults per month, venue one‐way door after midnight for more than 4 assaults per month and mandatory closing time of 2 AM for more than 8 per month) may have a greater impact, although resistance from the industry is likely to be more severe.

Providing feedback on injury data to nightlife venues is only likely to have an effect on people whose last drink was in a nightlife venue. This was just 23% of presentations that consumed alcohol in the last 12 h and presented during HAH. This contrasts with what would be expected from urban departments; for example, Benger et al. trialled a similar ED data sharing intervention and reported 53% of alcohol‐related presentations were coming from nightlife venues.^20^ Their study included cases outside of HAH, which likely lowered the frequency reported as having come from these venues. Rural patterns alcohol‐related presentations may be different from urban patterns in other ways. For instance, tourist seasons and public events have been reported as having a large influence on alcohol presentation patterns.^5^, ^21^ Elements of the Cardiff model, such as ED data collection informing a Violence Prevention Board, proved effective in this rural study. However, interventions other than nightclub feedback may be more effective outside urban areas. Rural Violence Prevention Boards could use their ED data to plan locally appropriate interventions such as changes to community policing, a tourist season education blitz, or partnerships with sporting clubs.

In addition, the findings from the present study clearly indicate that the majority of alcohol‐related attendances (just under three quarters) in this community involved the consumption of packaged liquor, and most injuries are occurring in private residences. While previous work has shown that the proportion of alcohol‐related presentations attributed to on‐premises consumption increased significantly during HAH,^21^ particularly at nightclub‐type venues, the majority of cases still originated from packaged liquor consumed in private residences. These data serve as an important piece of information for policy‐makers and future research trialling interventions, suggesting the need for greater understanding on how packaged liquor is impacting on the community, especially in the strong data showing a relationship between alcohol use and domestic violence.^22^, ^23^, ^24^

The frequencies from Table 2 indicate a potential predrinking for some alcohol‐related injuries, whereby attendants are likely consuming packaged liquor at private residences prior to attending licensed venues. A large‐scale Australian nightlife project has shown that approximately four‐fifths of patrons at nightlife venues drank alcohol before ‘going out’,^25^, ^26^ which is primarily motivated by price. The price disparity between off‐licence/on‐licence venues is a driving motivator for consumers to predrink packaged alcohol before attending nightlife venues and public events.

Finally, these findings again highlight the significant burden of alcohol use on health services in general and the emergency department in particular. They again suggest the need for greater intervention to reduce alcohol‐related harm in the community through the use of evidence‐based public health measures which reduce both acute and chronic disorders caused by excess alcohol consumption (such as a minimum unit price for alcohol and restrictions on advertising and decreased trading hours) and serve as an important reminder that a substantial proportion of the population continue to drink at levels above health guidelines (no more than 10 standard drinks per week and 4 standard drinks on any one occasion: https://www.nhmrc.gov.au/health‐advice/alcohol). This remains especially important for rural communities which continue to experience disproportionately higher levels of alcohol‐related harm than their urban counterparts.^5^, ^27^

### 4.1 Limitations

This study has several limitations. It is a single rural site, when other studies have shown that alcohol‐related presentations vary between towns of different sizes and location.^5^, ^28^ HAHs are a proxy for alcohol‐related harm, but alcohol‐associated injuries can occur at any time. Emergency departments represent the tip of the iceberg of alcohol‐related harm; only the more/most serious cases are taken to the hospital.

Further, although patients were asked about the number of drinks consumed in 12 h prior to their injury, no objective measure of intoxication was performed, such as breath alcohol concentration (BrAC). Also, in order to lessen the impact on ED practice, interrater reliability was not assessed for the present study and was not attempted using triage case notes due to the unreliable reporting of alcohol data in this form. Future research expanding on this work might attempt to incorporate interrater reliability and data quality measures.

### 4.2 Conclusions

Our study found that sharing last drinks data collected in the emergency department with a local violence prevention committee was associated with a small, but significant reduction in the rate of injury presentations compared with all ED presentations, despite most of the presentations being associated with packaged liquor and drinking at home. This intervention continues to have promise for reducing alcohol‐related harm, especially if the information networks are formalised and emergency department data can be included in licensing considerations and police proceedings.

## AUTHOR CONTRIBUTIONS

**Tim Baker:** Writing – original draft; writing – review and editing; investigation; funding acquisition; data curation. **Nicholas Taylor:** Writing – original draft; writing – review and editing; formal analysis; project administration. **Kate Kloot:** Investigation; funding acquisition; writing – review and editing; data curation. **Peter Miller:** Conceptualization; investigation; funding acquisition; methodology; writing – review and editing; supervision; project administration; writing – original draft; resources. **Diana Egerton‐Warburton:** Investigation; funding acquisition; writing – review and editing. **Jonathan Shepherd:** Investigation; funding acquisition; writing – review and editing.

## FUNDING INFORMATION

The trial described in this protocol has been funded by the National Health and Medical Research Council and St Vincent's Health Australia under the Partnership Project scheme (APP1113693).

## CONFLICT OF INTEREST STATEMENT

The authors have no conflict of interest to declare.

## ETHICS STATEMENT

Ethics approval was obtained from Deakin University (DUHREC 2017‐178) and the hospital involved in the study (SWHC 05/2017).
